# Associations between number of sick-leave days and future all-cause and cause-specific mortality: a population-based cohort study

**DOI:** 10.1186/1471-2458-14-733

**Published:** 2014-07-18

**Authors:** Emma Björkenstam, Gunilla Ringbäck Weitoft, Christina Lindholm, Charlotte Björkenstam, Kristina Alexanderson, Ellenor Mittendorfer-Rutz

**Affiliations:** 1 Department of Clinical Neuroscience, Division of Insurance Medicine, Karolinska Institutet, Berzelius väg 3, Stockholm SE-171 77, Sweden; 2 National Board of Health and Welfare, Stockholm, Sweden

**Keywords:** Mortality, Sick-leave days, Socioeconomic status, Gender, Morbidity, Inpatient care

## Abstract

**Background:**

As the number of studies on the future situation of sickness absentees still is very limited, we aimed to investigate the association between number of sick-leave days and future all-cause and cause-specific mortality among women and men.

**Methods:**

A cohort of 2 275 987 women and 2 393 248 men, aged 20–64 years in 1995 was followed 1996–2006 with regard to mortality. Data were obtained from linked authority-administered registers. The relative risks (RR) and 95% confidence intervals (CI) of mortality with and without a 2-year wash-out period were estimated by multivariate Poisson regression analyses. All analyses were stratified by sex, adjusting for socio demographics and inpatient care.

**Results:**

A gradually higher all-cause mortality risk occurred with increasing number of sick-leave days in 1995, among both women (RR 1.11; CI 1.07-1.15 for those with 1–15 sick-leave days to RR 2.45; CI 2.36-2.53 among those with 166–365 days) and men (RR 1.20; CI 1.17-1.24 to RR 1.91; CI 1.85-1.97). Multivariate risk estimates were comparable for the different causes of death (circulatory disease, cancer, and suicide). The two-year washout period had only a minor effect on the risk estimates.

**Conclusion:**

Even a low number of sick-leave days was associated with a higher risk for premature death in the following 11 years, also when adjusting for morbidity. This was the case for both women and men and also for cause-specific mortality. More knowledge is warranted on the mechanisms leading to higher mortality risks among sickness absentees, as sickness certification is a common measure in health care, and most sick leave is due to diagnoses you do not die from.

## Background

Today, the number of studies on risk factors for sickness absence has increased much, however, the number of studies on the future situation of sickness absentees is still very limited [1]. Most of the few studies so far conducted have focused on to what extent sickness absence can predict all-cause and cause-specific mortality in occupational [2,3] or population-based cohorts [2-8], with different follow-up periods. The risk of premature death varies somewhat with used measures of sickness absence, nevertheless, an excess rate of premature death was found in all studies [2-10]. There is a need to test those results in a larger, population-based cohort with a long follow-up period.

In welfare countries, to sickness certify patients is a common measure in healthcare, why more knowledge is warranted on associations between sickness absence and premature death. For instance, it is important to disentangle to what extent excess risks merely are due to a greater occurrence of morbidity among people who are sickness absent. There are several ways to measure morbidity, in research often self-reports are used [5,6,11]. Data on inpatient care, that is, being hospitalized, could be considered as data on more severe morbidity as well as on morbidity that has also been verified by physicians and therefore more valid. Also, the association between sickness absence and subsequent deaths might be due to the very disease that leads to the sickness absence. To account for that when trying to disentangle the associations between sickness absence and morbidity, sometimes a so called wash-out period is introduced, to exclude all deaths occurring in a near time frame, e.g., using a two-year wash out period [5].

Moreover, a great number of studies have shown gender differences in morbidity, in sickness absence, as well as in mortality [12-15]. There are several reasons to believe that the mechanisms leading to morbidity, to sickness absence, and to mortality might differ to some extent between the genders and that such aspects might not be visible when combining data for women and men, why gender-specific analyses are recommended [16-20].

The aim of this study was to investigate the associations between number of sick-leave days and future all-cause and cause-specific mortality among women and men, adjusting for morbidity and socioeconomic status, and also taking into account a two-year wash-out period for the relative risk of mortality.

## Methods

### Study population

A population-based prospective cohort study was conducted with an eleven-year follow up.

The cohort included all individuals aged 20–64 years, registered in Sweden on 31 December 1994 and 1995, and not on disability or old-age pension in 1995 (N = 4 669 235, 49% women). The cohort was identified from Statistics Sweden’s integrated population-based database for labor-market research (LISA). From this database also information regarding sick leave, disability pension, and other sociodemographic factors in 1995 was obtained. Further, date and cause of death was obtained from the Cause of Death Register, held by the National Board of Health and Welfare. The National Patient Register, also held by the National Board of Health and Welfare, was used to obtain information on inpatient care.

### Variables

All sick-leave days in 1995 reimbursed by the National Social Insurance Agency were included and categorized into five groups; 0 days (reference category), 1-15 days, 16-75 days, 76-165 days, and 166-365 days. From the age of 16 years, all Swedish residents with income from work, unemployment benefits, or student benefits are covered by the national sickness absence insurance regime and can be sickness absent with benefits if unable to work due to disease or injury. Benefits amount up to 80% of lost income. The first day of a sick-leave spell is a qualifying day, without any benefits. The first seven days can be self-certified; thereafter a physician certificate is needed. For those employed, the employer usually paid for the first 14 days of the sick-leave spell; those days are not registered in LISA and thus not included in this study. Unemployed people, if their morbidity lead to that they could not seek work, had sickness benefits paid by the Social Insurance Agency from the second day of the sick-leave spell. This means that for them also the shorter sick-leave spells were included. For most of the absentees, the category 1-15 days means that they had been sickness absent for 15-30 days.

Information about inpatient care was obtained for the years 1990-95, excluding hospitalization due to childbirth. The median number of days of inpatient care for those who were hospitalized was 5 days for the six-year period. Inpatient care days were categorized as; 0 days, 1-5 days, and more than 5 days.

In the analyses, age was used as a continuous variable and educational level was categorized into four groups: elementary school (9 years or less of schooling), secondary (10-12 years), university level (more than 12 years), or information missing. Type of place of residence was divided into a) large cities (Stockholm, Göteborg, and Malmö), b) middle-sized cities: places with more than 90 000 residents within 30 km from the municipal center, and c) rural municipalities (all other areas). Region of birth was categorized into Sweden, other Nordic country, other EU25, and other countries.

As outcome measure, we studied all-cause mortality for the years 1996-2006 as well as cause-specific mortality from circulatory diseases, cancer, and suicide, respectively.

### Statistical analyses

Multivariate analyses were conducted by Poisson regression with mortality as the dependent variable. The relative risk (RR) of mortality was estimated with 95% confidence interval (CI). Follow-up time was assessed by adding up the months the individuals were alive and residents of Sweden during the 11-years follow-up period 1996–2006. The associations between sick leave in 1995 and mortality were analyzed in five regression models. The reference group comprised individuals without sick-leave days in 1995. In model I, adjustments were made for age. In a second model (model II) we made additional adjustments for socio-demographic factors; i.e. educational level, type of place of residence, and region of birth. In model III, we adjusted for inpatient care in 1990-1995. Model IV included all covariates. In a fifth model, as an attempt to rule out that observed associations are not mainly due to an excess rate of sick leave shortly before death, a washout period of two years was used, that is, follow-up started in the third year after baseline (1998-2006). This included those who still lived 1 January 1998, conducting the same adjustment as in Model IV. Statistical analyses were carried out using the SAS software package, version 9.3. In accordance with the aim, all analyses were stratified by sex.

The project was approved of by the Regional Ethical Review Board of Stockholm (dnr 2007/762-31).

## Results

In this cohort of 4.7 million people of working ages, the mean age was about 40 years both among women and men. A slightly higher proportion of the men than the women had lower educational level (Table 1). About 70% of the women and 78% of the men had no inpatient care at all during 1990–1995. A higher rate of the women had had longer hospitalization. Also, a higher rate of the women had been sickness absent in 1995 and the mean number of sick-leave days during 1995 was 10.6 days in women and 8.0 days in men (not shown in table).

**Table 1:** Characteristics for the cohort of 4 669 235 women and men aged 20–64 and living in Sweden in 1995

	**Women (n = 2 275 987, 48.7%)**		**Men (n = 2 393 248, 51.3%)**		
	**n**	**%**	**n**	**%**	
Age (years)					
20-34	872 572	38.3	915 795	38.3	
35-44	544 934	23.9	573 897	24.0	
45-54	549 354	24.1	590 805	24.7	
55-64	309 127	13.6	312 751	13.1	
Educational level^1^					
Low	469 033	20.6	580 188	24.2	
Medium	1 145 377	50.3	1 181 081	49.4	
High	641 030	28.2	605 739	25.3	
Missing data	20 547	0.9	26 240	1.1	
Type of place of residency					
Rural/small	629 223	27.6	685 660	28.6	
Middle-sized city	820 991	36.1	867 944	36.3	
Large	825 773	36.3	839 644	35.1	
Region of birth					
Sweden	1 990 505	87.5	2 103 036	87.9	
Other Nordic	100 027	4.4	85 754	3.6	
EU25	49 742	2.2	50 887	2.1	
Other country	135 611	6.0	153 363	6.4	
Missing data	102	0.0	208	0.0	
No. of sick-leave days^2^ in 1995					
0	1 919 698	84.3	2 135 089	89.2	
1-15	137 973	6.1	96 883	4.0	
16-75	126 294	5.5	88 137	3.7	
76-165	43 758	1.9	31 834	1.3	
166-365	48 264	2.1	41 305	1.7	
No. of days with hospital in patient care in 1990-1995					
0	1 588 412	69.8	1 872 333	78.2	
1-5	343 370	15.1	302 106	12.6	
6+	344 205	15.1	218 809	9.1	

^1^Low = ≤9 years; Medium = 10–12 years; High = 13+ years;

^2^Days reimbursed by the Social Insurance Agency, usually from the 15^th^ day of a sick-leave spell.

The relative risks (RR) of all-cause mortality displayed a gradual increase with increasing number of sick-leave days in 1995 among both women and men (Table 2). In the age-adjusted models, women and men with most sick-leave days (166–365 days), had more than tripled mortality risks; women RR 3.48; 95% CI 3.37-3.60 and men RR 3.29; CI 3.20-3.39 in comparison with women and men, respectively, without any sick-leave days reimbursed by the Social Insurance Agency in 1995. Adjustments for socio-demographics did not substantially change the estimates. When inpatient care was taken into account (model III), the excess risks generally decreased, but the clear gradients remained. In the fully adjusted models (model IV), the risks decreased further. Using a “wash-out”-period of two years in model V, e.g., starting the follow-up 1998, decreased the excess risk to some extent regarding death risks associated with number of sick-leave days. However, evident associations remained with a pronounced gradient.

**Table 2:** Relative risk (RR) for all-cause mortality among women and men on sick leave with adjustments in four models and in a fifth model with two years wash-out period

**Sex**	**Sick-leave days**^**1**^	**Number of deaths**	**Model I**	**Model II**	**Model III**	**Model IV**	**Model V**
Women	0	35 833	1 (REF)	1 (REF)	1 (REF)	1 (REF)	1 (REF)
	1-15	3 086	1.24 (1.19-1.28)	1.23 (1.18-1.27)	1.11 (1.07-1.16)	1.11 (1.07-1.15)	1.11 (1.07-1.15)
	16-75	4 139	1.62 (1.57-1.67)	1.61 (1.56-1.66)	1.28 (1.24-1.32)	1.27 (1.23-1.32)	1.25 (1.21-1.30)
	76-165	2 242	2.42 (2.32-2.53)	2.39 (2.29-2.50)	1.81 (1.73-1.89)	1.79 (1.71-1.87)	1.60 (1.52-1.68)
	166-365	3 878	3.48 (3.37-3.60)	3.38 (3.27-3.50)	2.50 (2.42-2.59)	2.45 (2.36-2.53)	1.97 (1.89-2.05)
Men	0	63 407	1 (REF)	1 (REF)	1 (REF)	1 (REF)	1 (REF)
	1-15	4 556	1.47 (1.42-1.51)	1.41 (1.37-1.45)	1.24 (1.21-1.28)	1.20 (1.17-1.24)	1.20 (1.16-1.24)
	16-75	5 990	1.93 (1.88-1.99)	1.84 (1.80-1.89)	1.42 (1.38-1.46)	1.37 (1.33-1.41)	1.33 (1.29-1.37)
	76-165	3 192	2.66 (2.56-2.75)	2.52 (2.44-2.62)	1.71 (1.65-1.78)	1.65 (1.59-1.71)	1.53 (1.46-1.59)
	166-365	5 347	3.29 (3.20-3.39)	3.08 (3.00-3.17)	2.00 (1.95-2.07)	1.91 (1.85-1.97)	1.71 (1.65-1.77)

^1^Days reimbursed by the Social Insurance Agency, usually from the 15^th^ day of a sick-leave spell.

Model I: adjustment for age.

Model II: adjustment for age and education, type of place of residence, and region of birth.

Model III: adjustment for age, number of days in hospital (categorized).

Model IV: adjustment for age, number of days in hospital (categorized), education, type of place of residence, and region of birth.

Model V: model IV with two years wash-out period.

The RRs of mortality due to circulatory diseases are presented in Table 3. Although risk estimates were lower compared to RRs of all-cause mortality, a gradual increase with increasing number of sick-leave days among both women and men emerged. In a similar way, the risk estimates decreased after adjusting for several potential confounders. Even in the fully adjusted model (model IV), both women and men with more than 75 sick-leave days had a nearly 50% higher risk of premature death due to a circulatory disease during the follow-up period than women without such sick-leave days. Introducing a wash-out period did not change the adjusted RRs of premature death from circulatory diseases.

**Table 3:** Relative risk (RR) of premature death from circulatory diseases among women and men on sick leave with adjustments in four models and in a fifth model with two years wash-out period

**Sex**	**Sick-leave days**^**1**^	**Number of deaths**	**Model I**	**Model II**	**Model III**	**Model IV**	**Model V**
Women	0	6 366	1 (REF)	1 (REF)	1 (REF)	1 (REF)	1 (REF)
	1-15	520	1.20 (1.10-1.31)	1.19 (1.08-1.30)	1.08 (0.99-1.19)	1.07 (0.98-1.17)	1.11 (1.01-1.22)
	16-75	697	1.54 (1.42-1.67)	1.52 (1.41-1.64)	1.26 (1.16-1.37)	1.25 (1.16-1.36)	1.28 (1.18-1.40)
	76-165	319	1.92 (1.72-2.15)	1.89 (1.69-2.11)	1.43 (1.27-1.60)	1.42 (1.26-1.59)	1.40 (1.23-1.58)
	166-365	552	2.74 (2.51-2.99)	2.59 (2.37-2.82)	1.87 (1.71-2.05)	1.80 (1.64-1.97)	1.84 (1.67-2.03)
Men	0	19 632	1 (REF)	1 (REF)	1 (REF)	1 (REF)	1 (REF)
	1-15	1 331	1.38 (1.31-1.46)	1.32 (1.25-1.39)	1.18 (1.11-1.24)	1.13 (1.07-1.20)	1.15 (1.08-1.22)
	16-75	1 751	1.78 (1.70-1.87)	1.69 (1.61-1.78)	1.31 (1.24-1.38)	1.26 (1.19-1.32)	1.28 (1.21-1.35)
	76-165	866	2.24 (2.09-2.40)	2.12 (1.98-2.27)	1.41 (1.32-1.52)	1.36 (1.26-1.45)	1.37 (1.27-1.48)
	166-365	1 526	2.90 (2.75-3.05)	2.69 (2.56-2.84)	1.73 (1.63-1.83)	1.63 (1.54-1.72)	1.62 (1.53-1.72)

^1^Days reimbursed by the Social Insurance Agency, usually from the 15^th^ day of a sick-leave spell.

Model I: adjustment for age.

Model II: adjustment for age and education, type of place of residence, and region of birth.

Model III: adjustment for age, and number of days in hospital (categorized).

Model IV: adjustment for age, number of days in hospital (categorized), education, type of place of residence, and region of birth.

Model V: model IV with two years wash-out period.

The associations of number of sick-leave days with subsequent cancer-related death were similar (Table 4). Both women and men with at least one sick-leave day had a higher risk of premature death from cancer compared to those with no sick-leave days in 1995. In contrary to the analyses on death from circulatory diseases, the wash-out period decreased the adjusted RRs of death.

**Table 4:** Relative risk (RR) of premature death from cancer diagnoses among women and men on sick leave with adjustments in four models and in a fifth model with two years wash-out period

**Sex**	**Sick-leave days**^**1**^	**Number of deaths**	**Model I**	**Model II**	**Model III**	**Model IV**	**Model V**
Women	0	21 833	1 (REF)	1 (REF)	1 (REF)	1 (REF)	1 (REF)
	1-15	1774	1.17 (1.12-1.23)	1.16 (1.11-1.22)	1.08 (1.03-1.13)	1.07 (1.02-1.13)	1.08 (1.02-1.13)
	16-75	2318	1.49 (1.43-1.55)	1.48 (1.42-1.55)	1.19 (1.14-1.25)	1.19 (1.14-1.24)	1.14 (1.09-1.20)
	76-165	1347	2.38 (2.25-2.52)	2.37 (2.24-2.50)	1.91 (1.80-2.02)	1.90 (1.80-2.01)	1.60 (1.50-1.71)
	166-365	2331	3.42 (3.27-3.57)	3.38 (3.24-3.53)	2.81 (2.69-2.94)	2.78 (2.65-2.90)	1.93 (1.82-2.04)
Men	0	24 101	1 (REF)	1 (REF)	1 (REF)	1 (REF)	1 (REF)
	1-15	1461	1.24 (1.17-1.30)	1.21 (1.15-1.28)	1.14 (1.08-1.20)	1.11 (1.06-1.18)	1.10 (1.04-1.16)
	16-75	1937	1.60 (1.53-1.68)	1.57 (1.50-1.64)	1.34 (1.28-1.40)	1.31 (1.25-1.37)	1.20 (1.13-1.26)
	76-165	1089	2.28 (2.15-2.43)	2.23 (2.10-2.37)	1.76 (1.66-1.88)	1.72 (1.62-1.83)	1.40 (1.30-1.51)
	166-365	1813	2.79 (2.66-2.93)	2.70 (2.57-2.83)	2.19 (2.08-2.30)	2.12 (2.01-2.23)	1.59 (1.49-1.69)

^1^Days reimbursed by the Social Insurance Agency, usually from the 15^th^ day of a sick-leave spell.

Model I: adjustment for age.

Model II: adjustment for age and education, type of place of residence, and region of birth.

Model III: adjustment for age, and number of days in hospital (categorized).

Model IV: adjustment for age, number of days in hospital (categorized), education, type of place of residence, and region of birth.

Model V: model IV with two years wash-out period.

The analyses presented in Table 5 show that sick leave was associated with a higher risk of suicide in both women and men. The RR for suicide increased in a graded fashion with more sick-leave days in both women and men, even after adjustment for several potential confounding factors. Adjusting for inpatient care decreased the estimates significantly for both sexes. Taking a wash-out period of two years into consideration decreased the estimates somewhat for both women and men.

**Table 5:** Relative risk (RR) of premature death from suicide among women and men on sick leave, with adjustments in four models and in a fifth model with two years wash-out period

**Sex**	**Sick-leave days**^**1**^	**Number of deaths**	**Model I**	**Model II**	**Model III**	**Model IV**	**Model V**
Women	0	1 461	1 (REF)	1 (REF)	1 (REF)	1 (REF)	1 (REF)
	1-15	163	1.54 (1.31-1.81)	1.52 (1.29-1.79)	1.30 (1.11-1.53)	1.29 (1.10-1.52)	1.27 (1.06-1.52)
	16-75	256	2.65 (2.32-3.03)	2.60 (2.28-2.97)	1.85 (1.61-2.13)	1.84 (1.60-2.11)	1.70 (1.46-1.99)
	76-165	158	4.77 (4.05-5.62)	4.64 (3.93-5.47)	2.55 (2.14-3.02)	2.51 (2.12-2.99)	2.38 (1.95-2.89)
	166-365	257	7.13 (6.24-8.15)	6.77 (5.92-7.74)	2.90 (2.50-3.36)	2.83 (2.44-3.28)	2.64 (2.23-3.13)
Men	0	4 768	1 (REF)	1 (REF)	1 (REF)	1 (REF)	1 (REF)
	1-15	415	1.91 (1.73-2.12)	1.80 (1.62-1.99)	1.54 (1.39-1.70)	1.47 (1.33-1.62)	1.46 (1.31-1.64)
	16-75	497	2.54 (2.32-2.79)	2.36 (2.15-2.59)	1.69 (1.53-1.86)	1.62 (1.47-1.78)	1.60 (1.44-1.78)
	76-165	295	4.22 (3.75-4.75)	3.91 (3.47-4.40)	2.27 (2.01-2.57)	2.18 (1.93-2.46)	2.16 (1.88-2.48)
	166-365	439	4.99 (4.52-5.50)	4.57 (4.14-5.04)	2.28 (2.05-2.54)	2.20 (1.98-2.45)	1.97 (1.74-2.24)

^1^Days reimbursed by the Social Insurance Agency, usually from the 15^th^ day of a sick-leave spell.

Model I: adjustment for age.

Model II: adjustment for age and education, type of place of residence, and region of birth.

Model III: adjustment for age, and number of days in hospital (categorized).

Model IV: adjustment for age, number of days in hospital (categorized), education, type of place of residence, and region of birth.

Model V: model IV with two years wash-out period.

## Discussion

The results from this longitudinal study of 4.7 million women and men from the general population of working ages clearly demonstrated a gradual increase of all-cause and cause-specific premature death with increasing number of sick-leave days. Part of the mortality differences in the multivariate analyses was accounted for by differences in age, socio demographics, and particularly by morbidity, indicated by inpatient care. Results were similar for women and men. The two-year washout period had only a minor effect on the risk estimates.

Individuals having been sickness absent, also only for a few weeks, were found to have a higher risk for premature death, despite the fact that even individuals in the reference group might have been sickness absent later on, e.g., the year following the year of observation and despite the very long follow-up time of 11 years. More knowledge is warranted on these associations, regarding e.g. in different populations, time periods, and for different diagnoses. It might be argued that an association between sickness absence and premature death is expected as those on sick leave are that due to the underlying morbidity. However, the ill-health content of sick leave has often been questioned in the general debate [21,22]. Also, most sickness absences are due to musculoskeletal disorders – disorders that hardly ever are a cause of death, or due to minor mental disorders, which also very seldom are a cause of death with exception of suicide [13,21,23]. Many of the very short sick-leave spells are due to upper respiratory infections [13]. However, few of those are included in our data base due to the fact that the first 14 days of a sick-leave spell are paid by the employer. The main causes of death in Sweden as well as in other Western countries are circulatory disease and cancer – however, those diagnoses only stand for a few percentages of the sick-leave diagnoses [24].

Other hypotheses of the identified associations are that sick leave might be a marker of other factors, or that sick leave per se is a risk factor for morbidity or mortality. Different possible negative consequences of being sickness absent have been described in the literature, e.g. negative changes of life style (alcohol, exercise, diet, tobacco), of social interactions, of economy or work carrier, of the prognosis of the disease underlying the absence or of other diseases (e.g. depression) [1,25].

The higher risk of premature death with increasing number of sick-leave days is in line with previous studies [4,5,7,9]. As all these studies have somewhat different ways of defining sickness absence, the results from these studies cannot easily be compared. Still, findings from our study based on a very large population-based database, point in the same direction as previous studies and warrants follow ups using more specified research questions.

In our study, socio-demographic factors did not seem to explain much of the higher mortality risks. There is massive evidence on the associations between socio-economic status and mortality [12,26,27]. We used educational level as a proxy of socioeconomic status rather than income, in the attempt not to introduce an unnecessary gender bias (as women get less paid and a larger rate of women works part time). In general, people of lower socioeconomic status have higher risk for sickness absence [13,28], however, possible effects of sickness absence regarding premature death do not seem to differ between socioeconomic groups. The same can be said about gender; women have higher risks for becoming sickness absent; however, we in general found no gender differences regarding premature death among sickness absentees. It seems as the possible negative ‘effects’ of sickness absence do not vary between genders or socioeconomic groups.

Our results clearly indicate a higher RR for premature death due to circulatory diseases among women and men on sick leave, especially for those who had more than 15 sick-leave days in 1995, that is, mainly more than one months’ sick leave. This is in line with two previous studies based on occupational groups, finding that long-term sick leave is a risk factor for cardiovascular mortality [3,7]. Our results also correspond with another finding from these two studies [3,7], namely that sick leave was associated with cancer-related mortality.

We found an elevated suicide risk in women and men who had been on sick leave. The suicide risk increased in a graded manner as the number of sick-leave days increased, for both women and men. A few other studies have also found sick leave to be a risk factor for suicide [7,29,30]. In one of them, a case–control study with a lower number of women, sickness absence was a risk factor only for men [30]. It is well known that, in most Western countries, men have higher suicide rates than women [31,32]. However, here we studied the relative risks for suicide among sickness absent women and men, respectively, and found that those did not differ much between the genders.

### Strengths

The strengths of this study are the population-based, prospective cohort design, with a very long follow-up time, the very large number of included individuals, the high quality of the nationwide register data, e.g. regarding completeness and validity [33,34] and that there was no loss to follow up. The size of the cohort (4.7 millions) also provided sufficient power for gender-stratified analyses regarding rare outcomes such as suicide. As both the exposure and outcome were based on register data, there was no recall bias. Other strengths are that most of the sick-leave days as well as all hospitalizations were certified by a physician and that all individuals not at risk for sickness absence in 1995 were excluded, that is, those on disability pension.

### Limitations

The available information about health conditions was limited to morbidity leading to hospital care, which means that we were not able to account for all types of morbidity in the analyses. Hence, part of the higher risks may have been attributed to other types of impaired health among sick-listed people. On the other hand, it can be seen as an advantage that only the more severe morbidity was adjusted for, especially as morbidity seldom is accounted for in studies of associations between sick leave and premature deaths. That shorter sick-leave spells (<14 days) for employees could not be included can also be regarded both as a limitation and a strength. However, other studies have shown associations also with short-term sickness absence [7,10], which means that we rather have an under- than an over-estimation of results. Another limitation is that we could not exclude the sick-leave days from sick-leave spells shorter than 15 days - that is, days generally generated by unemployed people who had their sick-leave spells reimbursed by the Social Insurance Agency already from day 2. This means that there is an overrepresentation of unemployed among those in the category of 1-15 sick-leave days. However, most sickness absentees are working, and some of the unemployed do not have unemployment benefits- due to not having had a paid work lasting for 12 months or due to having been unemployed for too long time. This means that both in the reference group and in the group of fewer sick-leave days, there was a slight overrepresentation of unemployed. Future studies are needed to gain more knowledge on this. Despite the considerably large dataset including a number of important confounders, information on some potential confounders such as self-reports on health behavior including smoking and alcohol consumption could have been of importance. Moreover, the study was carried out using the year 1995 as baseline. Future studies are warranted to include cohorts based on other time periods and using other measures of sickness absence [35,36]. Moreover, the use of a two-year washout period can be questioned - both as being too long and as being too short - the effect of using different wash-out periods needs to be investigated in more detailed studies.

## Conclusions

There was a clear association between sickness absence and all-cause and cause-specific mortality, even for a relatively short number of sick-leave days, also when adjusting for morbidity. Moreover, the higher risks were about the same for women and men, although there are higher risks for women to become sickness absent. As sickness certification of patients is common in health care, more knowledge on possible mechanisms behind the results is warranted.

## Competing interests

The authors declare that they have no competing interests.

## Author’s contribution

EMR and KA originated the idea. GWR analysed the data in consultation with KA, CL, and EMR. EB wrote the first and subsequent drafts of the manuscript, with important intellectual input from all the co-authors. All authors contributed in designing the study and to the interpretation of the results and to the writing and approval of the final article.

## Pre-publication history

The pre-publication history for this paper can be accessed here:

http://www.biomedcentral.com/1471-2458/14/733/prepub
